# The impact of road traffic context on secondary task engagement while driving

**DOI:** 10.3389/fpsyg.2023.1139373

**Published:** 2023-04-03

**Authors:** Sandra Cuentas-Hernandez, Xiaomeng Li, Mark J. King, Oscar Oviedo-Trespalacios

**Affiliations:** ^1^QUT Faculty of Health, School of Psychology and Counselling, Queensland University of Technology (QUT), Brisbane, QLD, Australia; ^2^Delft Faculty of Technology, Policy and Management, Department of Values, Technology and Innovation, Delft University of Technology, Delft, Netherlands

**Keywords:** driver distraction, risky behavior, attention, multitask, human factors

## Abstract

**Introduction:**

Driver distraction has been recognized for a long time as a significant road safety issue. It has been consistently reported that drivers spend considerable time engaged in activities that are secondary to the driving task. The temporary diversion of attention from safety-critical driving tasks has often been associated with various adverse driving outcomes, from minor driving errors to serious motor vehicle crashes. This study explores the role of the driving context on a driver’s decision to engage in secondary activities non-critical to the driving task.

**Method:**

The study utilises the Naturalistic Engagement in Secondary Tasks (NEST) dataset, a complementary dataset derived from the SHRP2 naturalistic dataset, the most extensive naturalistic study to date. An initial exploratory analysis is conducted to identify patterns of secondary task engagements in relation to context variables. Maximum likelihood Chi-square tests were applied to test for differences in engagement between types of driver distraction for the selected contextual variables. Pearson residual graphs were employed as a supplementary method to visually depict the residuals that constitute the chi-square statistic.Lastly, a two-step cluster analysis was conducted to identify common execution scenarios among secondary tasks.

**Results:**

The exploratory analysis revealed interesting behavioral trends among drivers, with higher engagement rates in right curves compared to left curves, while driving uphill compared to driving downhill, in low-density traffic scenarios compared to high-density traffic scenarios, and during afternoon periods compared to morning periods. Significant differences in engagement were found among secondary tasks in relation to locality, speed, and roadway design. The clustering analysis showed no significant associations between driving scenarios of similar characteristics and the type of secondary activity executed.

**Discussion:**

Overall, the findings confirm that the road traffic environment can influence how car drivers engage in distracted driving behavior.

## 1. Introduction

Driver distraction involves a secondary task engagement while driving. Driver distraction has long been recognized as a major concern for road traffic safety. Regan et al. (2011) explain that driver distraction occurs when attention is diverted from safety-critical driving activities towards a competing activity. This temporary diversion of attention caused by the execution of competing activities at critical times has been recognized as a contributor to unwanted driving outcomes from minor errors to motor vehicle crashes (Dingus et al., 2016; Oviedo-Trespalacios et al., 2018b). Research has shown that drivers spend a considerable amount of time engaged in secondary activities while driving. A naturalistic study by Stutts et al. (2005) reported that drivers spent around 30% of the total motion time executing a distracting activity. Similarly, a more recent observational study (Young et al., 2019) reported that on average drivers engaged in nine secondary tasks per trip and spent 44.4% of the total driving time engaged in at least one secondary task. Over the years, the adoption of in-vehicle and portable technologies has added to an already long list of potential sources of distraction. Although order of prevalence may vary among studies, the most commonly distractions are usually conversing with passengers, eating and drinking, smoking, and manipulation of in-vehicle and portable electronic devices.

Driving behavior and decision-making can be modelled as a multi-level process. Michon (1985) proposed a hierarchical behavioral model for the driving task that explains action taking within three levels of resolution. The first two levels (operational and tactical) comprise factors prior to engagement and are key to identify the determinants of driving actions including any tasks considered as distractors. At the top level (strategic), the driver plans the journey including trip goals, route to take, time, and even defines which distracting activities are deemed acceptable to perform if the opportunity arises. At the tactical level, the driver negotiates the execution of a particular action depending on the overall demand of the ongoing driving task, the prevailing driving context circumstances and the expected demands of the new task. Lastly, operational level decisions are made post-engagement. Although these decisions are not determinants of the behavior, they come as a result of the actions taken. For example, reducing the speed to diminish overall workload and accommodate for the demands of new tasks being introduced. Between both pre-engagement decision making levels, the strategic level has received considerably more attention regarding the determinants behind secondary task engagement while driving compared to the tactical level. Psychosocial theories focused on planned behavior have been applied to explain drivers’ actions from an intentional or strategic point of view. On the other hand, research on tactical level decision making to engage in secondary tasks has been more limited. Considering several studies have provided evidence of discrepancies between an individual’s reported intentions and actual future behavior (Preece et al., 2018), analyzing the decision-making process at a tactical level is key to determine when and where engagement is more likely to take place.

In literature some evidence can be found that suggests drivers avoid engaging in secondary activities in high demanding driving scenarios (Liang et al., 2015; Kidd et al., 2016; Oviedo-Trespalacios et al., 2018a,b, 2019) while favoring those scenarios that they perceive to be less demanding. For instance, secondary task engagement has been reported to be more prevalent among drivers at standstill (i.e., stopped at controlled intersections) when compared to drivers in motion (Funkhouser and Sayer, 2012, Metz et al., 2014, Huisingh et al., 2015). Conditions that drivers seem to avoid include sharp curves, bad weather conditions, school areas, and high speeds. However, evidence to the contrary has also been presented. For instance, some research efforts, based on naturalistic data, have been unable to find associations between secondary task engagement and the characteristics of the driving environment including road surface conditions, time of drive, etc. (Stutts et al., 2001; Klauer et al., 2006). Furthermore, most research on the prevalence of secondary task while driving has concentrated on mobile phone use, with only a few studies examining the prevalence of other secondary tasks (Kidd et al., 2016).

This study further investigates the relationship between tactical components of the driving task and the decision to engage in driver distraction related activities using naturalistic data. Particular attention will be given to identifying what categories of secondary tasks share similar contextual characteristics for execution. It is hypothesized that secondary tasks with a similar level of complexity and resource demands will be executed in comparable driving environments.

## 2. Methods

### 2.1. Naturalistic engagement in secondary tasks dataset

The data used for this study was retrieved from the Naturalistic Engagement in Secondary Tasks (NEST) dataset. The NEST dataset is derived from the naturalistic driving data gathered by the Second Strategic Highway Research Program (SHRP2), a large-scale naturalistic study covering over 3,500 participants and six U.S. states over a three-year collection period. Data from the SHRP2 program was collected from instrumented vehicles equipped with a data acquisition system (DAS) with multiple channels for video and sensor data. Recorded data included information on variables such as vehicle speed, acceleration, lane position and location, as well as forward and rear camera views, and recordings of the drivers’ face and hands.

Publicly available SHRP2 data regarding secondary task engagement and its relation to crashes and near-crashes has a short time span of 6 s surrounding critical events. The main advantage of the NEST dataset over the SHRP2 data is that it allows for the study of engagement in secondary tasks for an extended period of time. The NEST dataset consists of close to a thousand excel files with both time-series and summary data related to secondary task engagement. Specifically, the NEST dataset was developed to provide extended detailed information on multiple factors related to secondary task engagement for time periods surrounding distraction-related safety-critical events (crash or near-crash) and baseline epochs with no safety-critical events associated. To select the trips to code from the SHRP2 dataset, all crash and near crash events preceded by engagement in a secondary task as a potential contributing factor were identified. A total of 236 safety-critical events were recognized and for each driver experiencing a safety-critical event, four baseline epochs were coded, for a total of 944 baseline epochs in the dataset.

In this study, only baselines epochs are of interest. Baselines are defined as 20 s epochs in which the driver was not involved in a crash or near crash. Contextual variables are coded in baseline epochs at different levels of resolution. Both summary and time-series data are used to describe contextual characteristics during the period of time to be analyzed.

**Figure 1:**
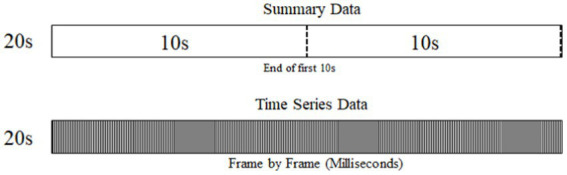
Data resolution levels.

Time-series data are coded using frame-by-frame analysis and are recorded for every millisecond in time. Summary data describes the baseline epochs at an event level. The reduced summary data describes the 20s period in two levels of resolution. Variables such as weather are coded once for the entire 20s period while variables such as traffic density are coded at the end of every 10 s of the event (i.e., two times per event) as seen in Figure 1.

Table 1 lists the contextual variables of interest along with their respective levels of resolution. It is important to highlight that only variables that were possible to accurately extract from the dataset were included.

**Table 1:** Context variables definitions and resolution levels.

Context variable	Definition	Level of resolution
Traffic Flow	Describes the roadway design (e.g., one-way, divided highway, etc.) at the end of the 10s block	Summary data (10s blocks)
Travel Lanes	Identifies the number of travel lanes at the end of the 10s block	Summary data (10s blocks)
Traffic Density	Describes the density of traffic (level of service) at the end of the 10s block	Summary data (10s blocks)
Intersections entered	Number of controlled intersections entered during the 10s block	Summary data (10s blocks)
Alignment	Roadway alignment (e.g., straight, curve left, etc.) at end of the 10 s block	Summary data (10s blocks)
Road Grade	Roadway grade (e.g., level, grade up, grade down, etc.) at the end of the 10s block	Summary data (10s blocks)
Locality	Description of the surrounding area (e.g., residential, business, interstate, etc.) the vehicle is in at the end of the 10s block	Summary data (10s blocks)
Lighting	Description of ambient lighting at the end of the 10s block (e.g., dawn, daylight, dusk, etc.)	Summary data (10s blocks)
Time bins	Time Bin (local to the data collection site)	Time series
Speed (GPS/speedometer)	Vehicle speed calculated from change in GPS position or indicated by speedometer.	Time series

### 2.2. Further data reduction

Note that it is possible to identify one, multiple or none of the secondary tasks during a baseline epoch. In addition, baseline epochs may not contain the point of initiation of a secondary task.

**Figure 2:**
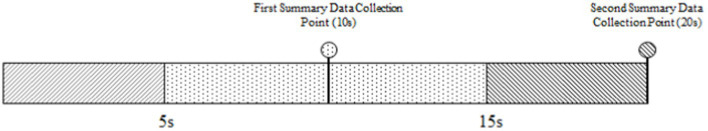
Times series data approximation.

To obtain the final dataset, additional filtering and data reduction is performed, the process is as follows:

1. Baseline events containing at least one secondary task are filtered. This filter eliminated 208 baselines events with no secondary tasks associated, for a remaining total of 736 baseline events.
2. The remaining baselines events are filtered to include only those in which the engagement point of the secondary task is recorded in the 20s epoch and those where there is not a simultaneous occurrence of more than one secondary task.
3. For analysis purposes, similar secondary tasks are grouped together into new categories as shown in Table 2.
4. As summary data is recorded in 20s or 10s blocks, it is necessary to match summary data to a time series level of resolution at the point of secondary task initiation. The logic is as follows: All secondary tasks starting between 5 and 14 s are allocated the summary data recorded at the end of the first 10s block, while all secondary tasks starting from the 15 s mark are allocated the summary data recorded at the end of the second 10s block. All secondary tasks starting within the first 5 s of the 20s window are excluded as the summary data collection points are distant from the occurrence of the secondary task (Figure 2).

**Table 2:** Secondary task categorization.

Task Category	Secondary Tasks
Mobile phone use	Texting
	Holding
Grooming	Applying makeup
	Removing/adjusting jewelry
	Combing/brushing/fixing hair
	Other personal hygiene
Passenger	Talk to Passenger
Dancing	Dancing
Adjusting Internal Device	Adjusting/monitoring climate control
	Adjusting/monitoring other devices integral to vehicle
	Adjusting/monitoring radio

### 2.3. Statistical analysis

The statistical analysis is divided in two phases. First, a descriptive statistical analysis is performed to describe patterns of engagement of the selected secondary tasks under different contextual variables (Table 3). A maximum likelihood Chi-square test was applied to test for differences in engagement between types of driver distraction for the selected contextual variables. A *p*-value of <0.05 was considered statistically significant. Pearson residuals graphs were used to visualize the cells contributing the most to the Chi-square score. The residuals quantify the difference between the observed data proportions and the expected data proportions under the assumption that there is no relationship between the row and column categories of the table. The resulting graphical matrix contains a dot whose size reflects the relative magnitude of association it contains, and the color of the dot differentiates between positive (blue) and negative (red) associations.

**Table 3:** Frequency of engagement in secondary tasks.

Variables		Cellphone use (Texting)		Cellphone use (Holding)		Internal device		Passenger interaction		Dancing		Grooming		G	*p*
		*n* (53)	%	*n* (22)	%	*n* (67)	%	*n* (81)	%	*n* (20)	%	*n* (28)	%		
Locality	Business/industrial	19	35.85%	8	36.36%	19	28.36%	34	41.98%	10	50.00%	7	25.00%	43.508	0.0123
	Bypass/divided highway with traffic signals	2	3.77%	0	0.00%	5	7.46%	14	17.28%	3	15.00%	2	7.14%		
	Interstate/bypass/divided highway with no traffic signals	18	33.96%	6	27.27%	18	26.87%	15	18.52%	1	5.00%	4	14.29%		
	Moderate Residential	8	15.09%	1	4.55%	14	20.90%	10	12.35%	4	20.00%	11	39.29%		
	Open residential	3	5.66%	3	13.64%	2	2.99%	3	3.70%	1	5.00%	1	3.57%		
	Special Zones (School, Church, Construction Zone)	3	5.66%	4	18.18%	9	13.43%	5	6.17%	1	5.00%	3	10.71%		
Alignment	Curve left	3	5.66%	1	4.55%	2	2.99%	8	9.88%	1	5.00%	1	3.57%	5.7128	0.8388
	Curve right	5	9.43%	2	9.09%	7	10.45%	5	6.17%	1	5.00%	1	3.57%		
	Straight	45	84.91%	19	86.36%	58	86.57%	68	83.95%	18	90.00%	26	92.86%		
Intersection	0	45	84.91%	18	81.82%	57	85.07%	69	85.19%	18	90.00%	21	75.00%	2.3415	0.8001
	1	8	15.09%	4	18.18%	10	14.93%	12	14.81%	2	10.00%	7	25.00%		
LOS	A	31	58.49%	14	63.64%	39	58.21%	56	69.14%	11	55.00%	16	57.14%	13.219	0.9737
	B	16	30.19%	6	27.27%	19	28.36%	20	24.69%	6	30.00%	11	39.29%		
	C	4	7.55%	2	9.09%	6	8.96%	4	4.94%	2	10.00%	1	3.57%		
	D	1	1.89%	0	0.00%	1	1.49%	1	1.23%	1	5.00%	0	0.00%		
	E	1	1.89%	0	0.00%	1	1.49%	0	0.00%	0	0.00%	0	0.00%		
	F	0	0.00%	0	0.00%	1	1.49%	0	0.00%	0	0.00%	0	0.00%		
No. Lanes	0	0	0.00%	1	4.55%	2	2.99%	1	1.23%	0	0.00%	0	0.00%	46.726	0.08888
	1	6	11.32%	0	0.00%	5	7.46%	4	4.94%	0	0.00%	1	3.57%		
	2	21	39.62%	8	36.36%	29	43.28%	40	49.38%	7	35.00%	9	32.14%		
	3	9	16.98%	3	13.64%	7	10.45%	21	25.93%	4	20.00%	8	28.57%		
	4	9	16.98%	7	31.82%	14	20.90%	6	7.41%	3	15.00%	2	7.14%		
	5	7	13.21%	3	13.64%	7	10.45%	8	9.88%	3	15.00%	6	21.43%		
	6	0	0.00%	0	0.00%	2	2.99%	1	1.23%	2	10.00%	2	7.14%		
	7	1	1.89%	0	0.00%	1	1.49%	0	0.00%	1	5.00%	0	0.00%		
Roadgrade	Grade Down	1	1.89%	1	4.55%	2	2.99%	6	7.41%	0	0.00%	2	7.14%	10.093	0.8138
	Grade Up	5	9.43%	3	13.64%	5	7.46%	10	12.35%	2	10.00%	3	10.71%		
	Hillcrest	2	3.77%	1	4.55%	2	2.99%	1	1.23%	0	0.00%	0	0.00%		
	Level	45	84.91%	17	77.27%	58	86.57%	64	79.01%	18	90.00%	23	82.14%		
Lighting	Darkness, lighted	9	16.98%	5	22.73%	10	14.93%	9	11.11%	3	15.00%	2	7.14%	20.482	0.4282
	Darkness, not lighted	4	7.55%	5	22.73%	8	11.94%	4	4.94%	1	5.00%	2	7.14%		
	Dawn	0	0.00%	0	0.00%	1	1.49%	0	0.00%	0	0.00%	0	0.00%		
	Daylight	37	69.81%	10	45.45%	44	65.67%	66	81.48%	16	80.00%	23	82.14%		
	Dusk	3	5.66%	2	9.09%	4	5.97%	2	2.47%	0	0.00%	1	3.57%		
Timebins	0 (12–3 AM)	2	3.77%	6	27.27%	6	8.96%	9	11.11%	1	5.00%	2	7.14%	35.939	0.2101
	1 (3−6 AM)	0	0.00%	0	0.00%	3	4.48%	2	2.47%	0	0.00%	0	0.00%		
	2 (6−9 AM)	6	11.32%	2	9.09%	8	11.94%	9	11.11%	1	5.00%	1	3.57%		
	3 (9 AM −12 PM)	4	7.55%	0	0.00%	8	11.94%	13	16.05%	4	20.00%	5	17.86%		
	4 (12–3 PM)	13	24.53%	4	18.18%	8	11.94%	12	14.81%	5	25.00%	6	21.43%		
	5 (3–6 PM)	8	15.09%	4	18.18%	9	13.43%	21	25.93%	4	20.00%	4	14.29%		
	6 (6–9 PM)	11	20.75%	6	27.27%	12	17.91%	9	11.11%	3	15.00%	4	14.29%		
	Null	9	16.98%	0	0.00%	13	19.40%	6	7.41%	2	10.00%	6	21.43%		
Roadway	Divided (median strip or barrier)	34	64.15%	13	59.09%	27	40.30%	42	51.85%	7	35.00%	8	28.57%	28.51	0.01859
	No lanes	3	5.66%	1	4.55%	3	4.48%	1	1.23%	0	0.00%	0	0.00%		
	Not divided	13	24.53%	8	36.36%	33	49.25%	34	41.98%	13	65.00%	19	67.86%		
	One-way traffic	3	5.66%	0	0.00%	4	5.97%	4	4.94%	0	0.00%	1	3.57%		
Speed	(0,22)	1	1.89%	2	9.09%	5	7.46%	4	4.94%	0	0.00%	0	0.00%	46.253	0.005997
	(22,44)	6	11.32%	4	18.18%	2	2.99%	19	23.46%	6	30.00%	4	14.29%		
	(44,66)	9	16.98%	3	13.64%	24	35.82%	19	23.46%	4	20.00%	6	21.43%		
	(66,88)	10	18.87%	5	22.73%	5	7.46%	16	19.75%	5	25.00%	8	28.57%		
	(88,110)	11	20.75%	4	18.18%	16	23.88%	14	17.28%	2	10.00%	2	7.14%		
	(110,132)	7	13.21%	3	13.64%	2	2.99%	4	4.94%	1	5.00%	2	7.14%		
	null	9	16.98%	1	4.55%	13	19.40%	5	6.17%	2	10.00%	6	21.43%		

Next, a two-step cluster analysis procedure was used to analyze the influence of contextual factors on secondary task engagement while driving. The procedure is an exploratory tool that groups cases (objects to be clustered) of data based on homogeneous responses to several variables (attributes). The objective of the clustering analysis is to determine if any of the secondary tasks shared similar contextual characteristics for execution. The analysis is based on the idea that tasks executed at the same time compete for a shared pool of multiple resources as suggested by several attentional resource theories. As a result, the extent in which the driving task and any secondary tasks performed simultaneously are able to allocate available resources will determine if the driver deems their execution as having non-significant cross-task interference. Based on this, driving scenarios with comparable demands would allow for similar levels of free resources to be allocated to other tasks, and therefore, secondary tasks of similar characteristics would be able to be accommodated in driving contexts with comparable demands. The analysis is conducted in IBM SPSS Statistics (version 27) and as indicated by its name; it consists of two major steps.

Step 1. In the first step, a sequential clustering approach is used to create many subclusters. The process consists of constructing a Cluster Features (*CF*) tree of the cases. After the initial case is placed at the root of the tree, then successive decisions on whether the next case joins an already formed cluster or a new cluster are made based on a similarity measure. If all attributes are continuous, cases are grouped in the subcluster using the smallest Euclidean distance. To handle continuous and categorical variables, the log-likelihood distance measure, a probability-based distance, is used. Cases are grouped in the cluster with the highest likelihood measure. To implement this measure, continuous variables are assumed to have a normal distribution while categorical variables are assumed to have a multinomial distribution. Additionally, all variables are assumed to be independent. However, the two-step clustering procedure has proven to be robust to violations of independence and distributional assumptions.

Step 2. In the second step, an agglomerative hierarchical clustering algorithm is used to merge the subclusters stepwise into the desired the number of clusters. The process starts by selecting a starting cluster for each of the sub-clusters formed in Step 1. Clusters are compared, and the pair of clusters that yield the smallest distance are merged. The measure of the distance between two clusters corresponds to the decrease in log-likelihood when the two clusters are merged. The merging process is repeated recursively until the final number of clusters is reached. The final number of clusters can be a previously fixed number, or it can be automatically determined by choosing between two possible options. Either the Schwarz’s Bayesian Criterion (BIC) or the Akaike Information Criterion (AIC) can be determined as the clustering criterion. The optimal value is found by comparing the values of the chosen clustering criterion across different clustering solutions.

For validation purposes, maximum likelihood Chi-square tests were carried out after cluster formation. The tests assessed whether significant differences were present for contextual variables between- clusters. If differences were not significant, the cluster analysis was repeated maintaining the contextual variables found to be of relevance in cluster partitioning (Table 4).

**Table 4:** Context variables between-cluster differences.

	Variables	Cluster 1	Cluster 2	G	DF	*p*-value
Locality	Business/industrial/Urban	59	39	169.36	5	<2.2e-16
	Bypass/divided highway with traffic signals	4	22			
	Interstate/bypass/divided highway with no traffic signals	5	57			
	Moderate residential	47	0			
	Open residential	13	0			
	Special zone	23	2			
Alignment	Curve left	7	9	0.98356	2	0.6115
	Curve right	12	9			
	Straight	132	102			
Intersection	0	113	115	25.162	1	5.27E-07
	1	38	5			
LOS	A	118	49	53.728	5	2.38E-10
	B	30	48			
	C	1	18			
	D	1	3			
	E	0	2			
	F	1	0			
No. Lanes	0	0	4	66.98	7	6.01E-12
	1	16	0			
	2	82	32			
	3	20	32			
	4	8	33			
	5	19	15			
	6	4	3			
	7	2	1			
Roadgrade	Grade Down	9	3	4.246	3	0.2361
	Grade Up	15	13			
	Hillcrest	5	1			
	Level	122	103			
Lighting	Darkness, lighted	26	12	9.7481	4	0.04489
	Darkness, not lighted	18	6			
	Dawn	1	0			
	Daylight	99	97			
	Dusk	7	5			
Timebins	0 (12–3 AM)	21	5	25.33	6	0.0002966
Timebins	1 (3−6 AM)	1	4			
	2 (6−9 AM)	6	21			
	3 (9 AM −12 PM)	17	17			
	4 (12–3 PM)	41	23			
	5 (3–6 PM)	31	27			
	6 (6–9 PM)	34	23			
Roadway	Divided (median strip or barrier)	16	115	256.91	3	2.20E-16
	No lanes	4	4			
	Not divided	120	0			
	One-way traffic	11	1			
Speed	(0,22)	5	7	144.02	5	2.20E-16
	(22,44)	32	9			
	(44,66)	74	14			
	(66,88)	39	23			
	(88,110)	1	48			
	(110,132)	0	19			

## 3. Results

### 3.1. Descriptive analysis

General descriptives of the data analyzed in the present study are presented in Table 3. Detail analysis are presented in the following subsections.

#### 3.1.1. Locality

Occurrences of secondary task engagement were more prevalent in Business/Industrial localities defined as areas where any type of business or industrial structure is present. Overall, around 35.8% of all secondary tasks started in Business/Industrial localities. Other common localities for secondary task engagement were moderate residential areas (multiple houses or apartment buildings are present) with close to 17.8% of occurrences and the Interstate/bypass/divided highway with no traffic signals category with around 22.9% of occurrences.

The association between locality and type of secondary task was significant according to the maximum likelihood Chi-square test (G = 55.891, *p* < 0.05). For all secondary tasks, except grooming, engagement was more frequent in Business/Industrial localities. Passenger interactions and dancing returned the highest rates of occurrences in Business/Industrial localities with 42 and 50% of their total occurrences executed in this category, respectively. Grooming was more common in moderate residential areas.

Engagement rates for mobile phone use and internal device use were higher for the Interstate/bypass/divided highway with no traffic signals category when compared to the engagement rates of the remaining secondary activities within this category.

**Figure 3:**
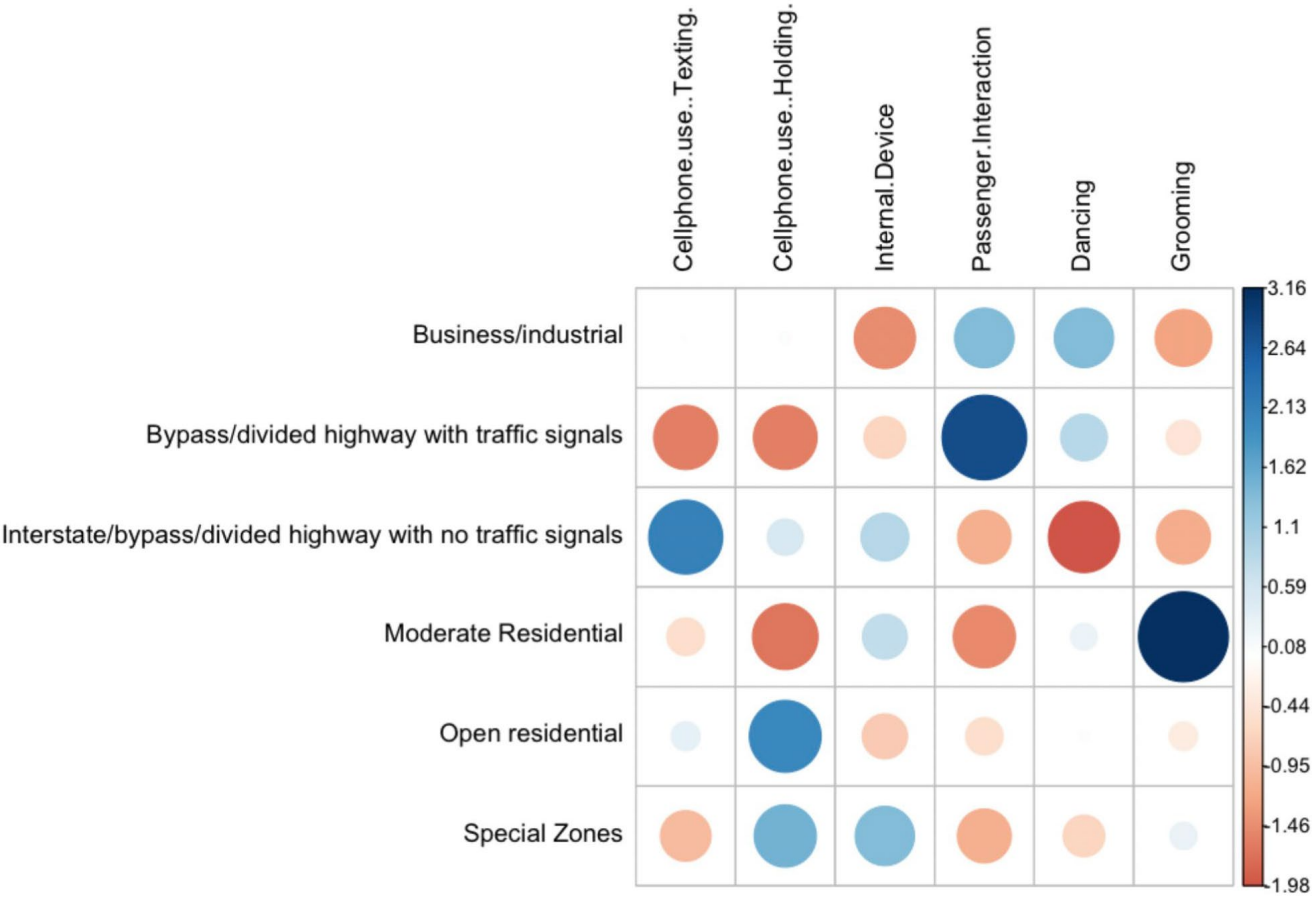
Pearson residuals for locality and secondary task type.

Pearson residuals (Figure 3) visually confirmed some of the findings including a higher engagement than expected in grooming tasks in moderate residential localities. Other associations that were noticeable were a higher engagement in passenger interaction in highways with traffic signals and a lower engagement for tasks such as dancing, grooming and passenger interactions tasks in highways with no traffic signals.

#### 3.1.2. Roadway alignment

All secondary activities were more commonly executed in straight segments as expected given that other road configurations are not as common during the driving task. Engagement rates in right curves were close to doubled when compared to engagement rates in left curves for mobile phone use (both texting and holding) and tripled for internal device use. Dancing and grooming were reported to have similar engagement rates for both right and left curves. Only passenger interactions showed a higher engagement rate in left curves when compared to right curves which accounted for a third of the total occurrences in curved segments.

#### 3.1.3. Level of service

The level of service variable was used to describe the density of the traffic during the driving task. Six different traffic density levels, from A to F, were defined based on the number of vehicles, and the ability of the driver to select the driving speed. For all secondary tasks, engagement decreased as traffic density increased. Levels A and B were shown to have the higher rates of engagement with 61.6 and 28.8% of total secondary task occurrences, respectively. Level A is defined as free flow conditions with drivers unaffected by the presence of others and unrestricted manoeuvrability and ability to select desired speeds. Level B comprises stable flow conditions where influence from other users starts to be noticeable, while desired speeds are relatively unaffected, manoeuvrability within the traffic stream is slightly diminished when compared to free flow conditions. Engagement in the remaining levels of service was shown to decrease as traffic density levels increased.

#### 3.1.4. Roadgrade

As expected, given predominant road configurations, all secondary activities were more commonly executed in road segments with a level grade (83%). Higher rates of engagement were found while drivers circulated in grade up roads when compared to grade down roads for all secondary activities considered.

#### 3.1.5. Intersections entered

The variable contains the number of controlled intersections entered during the recorded event. When comparing secondary tasks, the higher rate of engagement at intersections was reported for grooming tasks while the lower rate of engagement was reported for dancing.

#### 3.1.6. Ambient lighting

All secondary activities were more commonly executed under daylight conditions with 72.3% of total occurrences. Engagement under dark ambient lighting, both with lighted and non-lighted roads, accounted for close to 23% of total occurrences. For secondary tasks such as texting, passenger interactions and dancing, engagements rates under darkness conditions with road lights more than doubled engagement rates under darkness conditions in non-lighted roads. Holding a mobile phone, internal device use and grooming recorded similar engagement rates regardless of the presence of road lights. Dusk and dawn reported very low rates of engagement, however, in general, engagement rates during dusk were higher.

#### 3.1.7. Time bins

Engagement in secondary tasks while driving was higher during the afternoon periods compared to morning periods. The highest peaks of engagement in secondary task occurred during the three time bins comprising the 12–9 PM time window with an even distribution. About 16.2% of secondary task occurrences started between 6 and 9 PM, where holding a mobile phone and internal device use reported the highest number of occurrences for their category during the day. Texting, dancing and grooming reached their peak during the 12–3 PM time period. Mobile phone use rates were higher between 6 and 9 PM when compared to the remaining activities. No secondary task occurrences were recorded between 9 PM and midnight for all activities in consideration.

#### 3.1.8. Number of lanes

All secondary activities were more commonly executed while circulating in two-lane roads with about 42% of the total number of occurrences. In general, engagement was more prevalent between 2 to 4 lane roads. Engagement in three lane roads constituted around 19.2% of occurrences while engagement in four-lane roads accounted for 15.12% of total occurrences. For internal device use and holding a mobile phone, the rates of engagement close to double in four-lane roads when compared to three-lane roads. Texting and dancing displayed similar rates of engagement for both 3-lane and 4-lane roads, while passenger interactions and grooming displayed lower rates of engagement in four-lane roads when compared to three lane roads. Interestingly, the grooming engagement rate in 5-lane roads was considerably higher compared to all other activities.

#### 3.1.9. Roadway design

**Figure 4:**
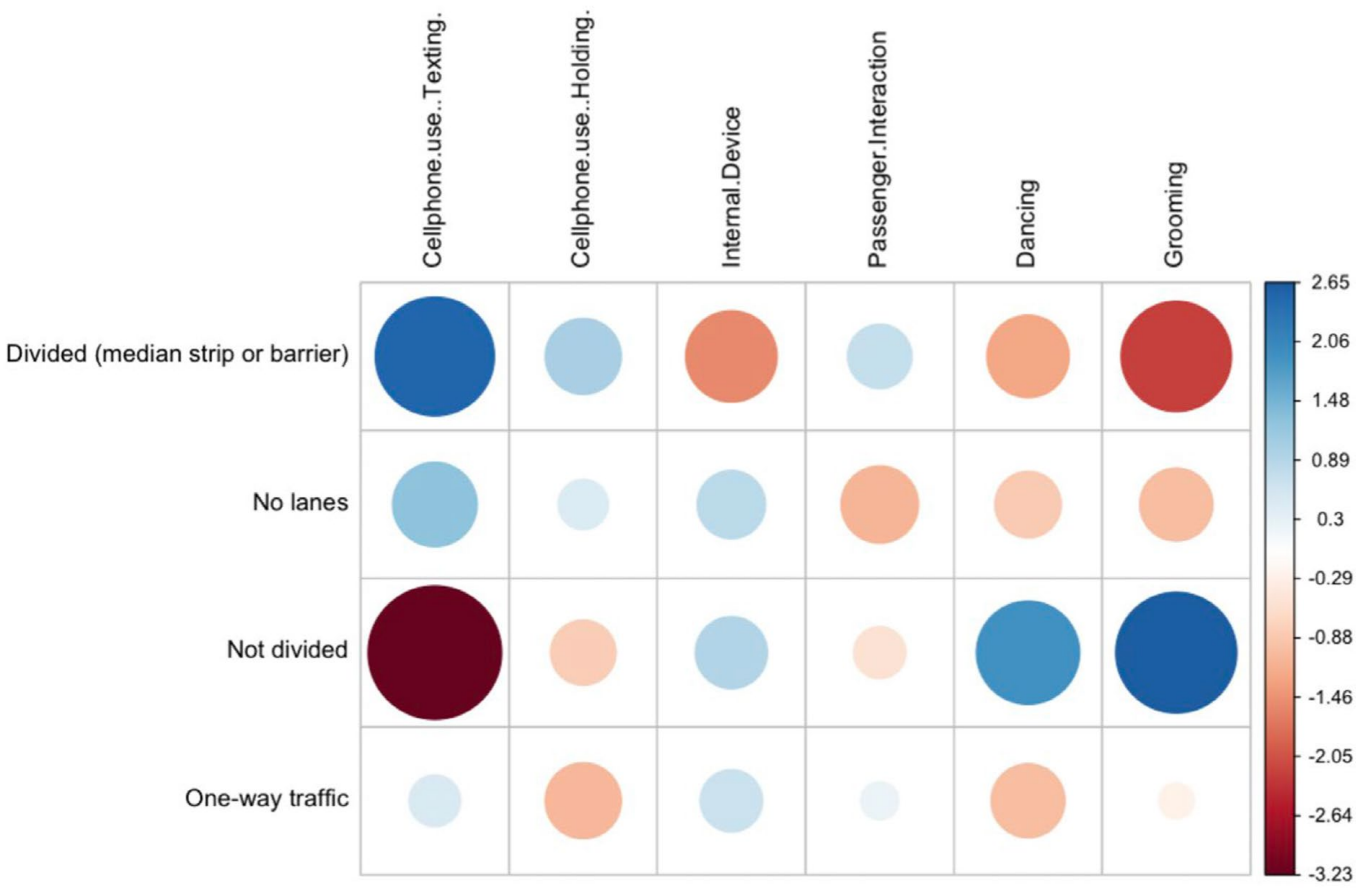
Pearson residuals for roadway design and secondary task type.

Engagement in secondary tasks while driving was similar for divided (median strip or barrier) and non-divided roads with 48.3 and 44.2% of total occurrences, respectively. The association between roadway design and type of secondary task was significant according to the maximum likelihood Chi-square test (G = 28.51, *p* < 0.02). When analyzing by secondary task type, mobile phone use rates in divided roads were considerably higher when compared to engagement rates in non-divided roads, as visually corroborated by the Pearson residuals graph in Figure 4. Engagement rates were also higher for passenger interactions in divided roads when compared to non-divided roads, however, the difference was smaller. All other secondary tasks were more prevalent in non-divided roads than in divided roads. Dancing and grooming engagement rates in non-divided roads close to double engagement rates in divided roads. Internal device use was also higher in non-divided roads, but the difference was considerably smaller. One-way traffic roads registered low engagement rates for all secondary activities considered.

#### 3.1.10. Speed

The mean speed of engagement for most secondary tasks ranged from 60 to 70 km/h. The association between speed and type of secondary task was significant according to the maximum likelihood Chi-square test (G = 33.877, *p* < 0.03).

**Figure 5:**
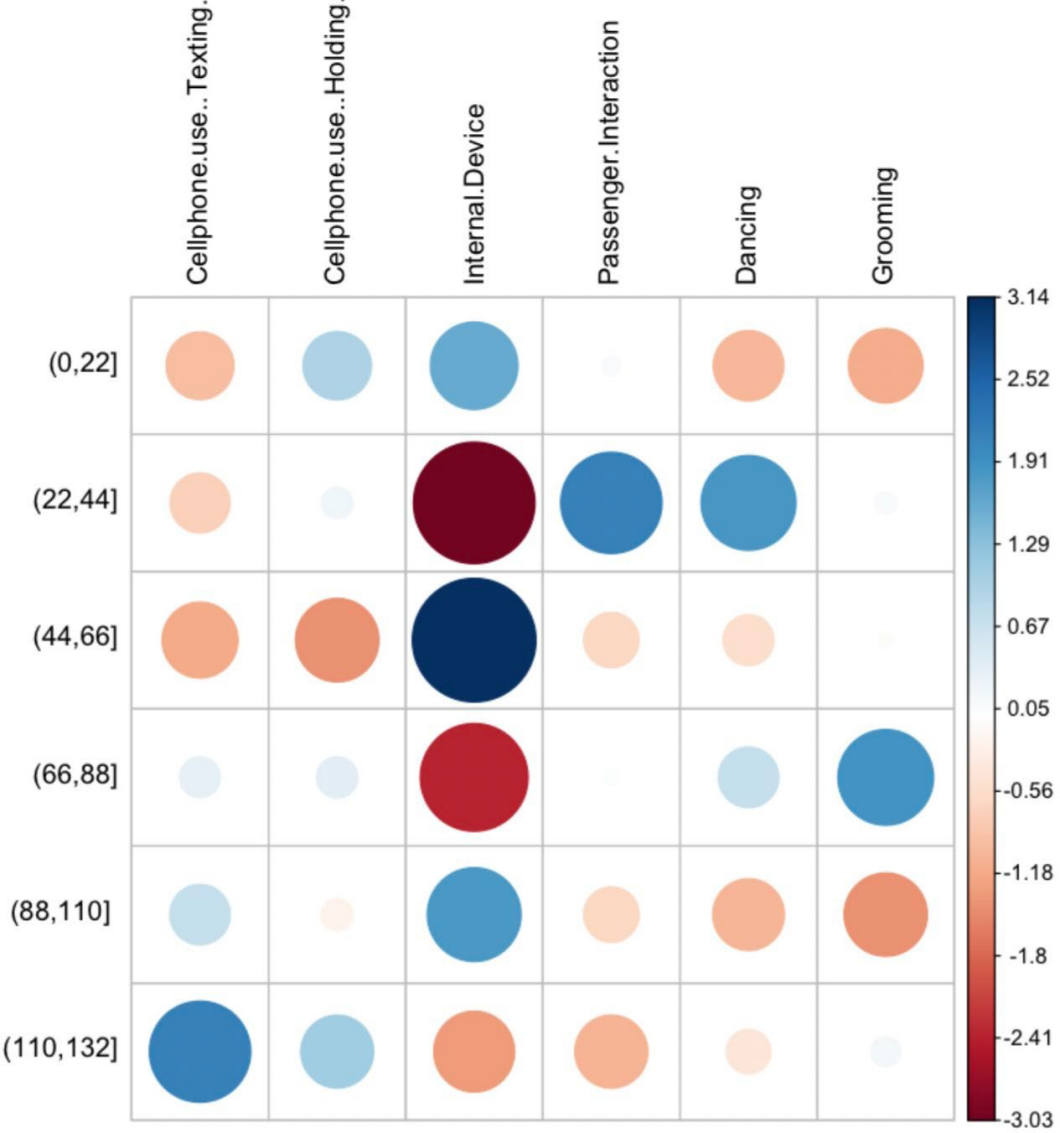
Pearson residuals for speed and secondary task type.

Engagement in texting occurred at higher rates between 45 and 110 km/h with an increasing trend between that range. Rates of engagement were more evenly distributed for mobile phone holding with higher engagement rates between 0 and 45 km/h when compared to texting. Both mobile phone activities showed higher proportion of engagement above 110 km/h when compared to other activities (Figure 5).

For internal device use, two peaks of use are noticeable, one between 45 and 70 km/h and another between 90 and 110 km/h with a gap of lower occurrences in between. Occurrences in both peaks are noticeably higher compared to other secondary tasks in the same speed ranges. Internal device use between 20–45 km/h and 65–90 km/h was also noticeably lower when compared to other secondary tasks in the same speed bins. Most occurrences of passenger interactions took place between 20 and 110 km/h with a fairly even distribution with a slightly decreasing trend, engagement rates below 20 km/h and above 119 km/h were markedly lower in comparison.

Engagement in grooming tasks increased steadily between 0 and 90 km/h with the lowest rates of engagement for speeds above 90 km/h. Dancing tasks seem to favor lower speeds with most occurrences below 90 km/h. Engagement rates between 20–45 km/h were higher when compared to other secondary tasks.

### 3.2. Cluster analysis

Two-step cluster analysis was carried out in 271 occurrences of secondary task engagement while driving to identify common scenarios for execution. The final number of clusters was determined in accordance with the Schwarz’s Bayesian criterion (BIC). Two context variables were eliminated during the validation phase, alignment and road grade (Table 4).

**Figure 6:**
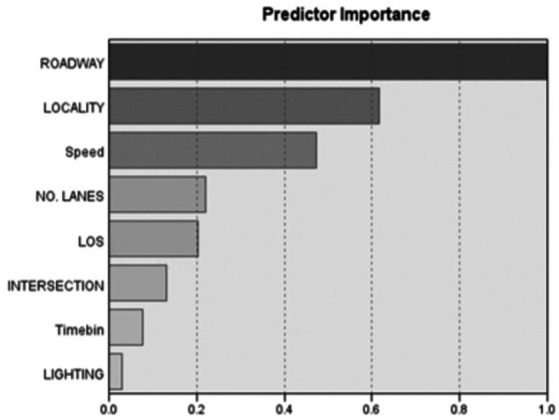
Predictor importance for two-step clustering.

Two distinctive clusters were identified, Cluster 1 comprising 55.7% of secondary task occurrences while Cluster 2 comprised 44.3% of secondary task occurrences. The Silhouette measure which contrasts the average distance to elements within the same cluster yielded a value of 0.3 (fair) while the ratio of sizes from largest to smallest cluster yielded a value of 1.26. Predictor importance is displayed in Figure 6.

**Figure 7:**
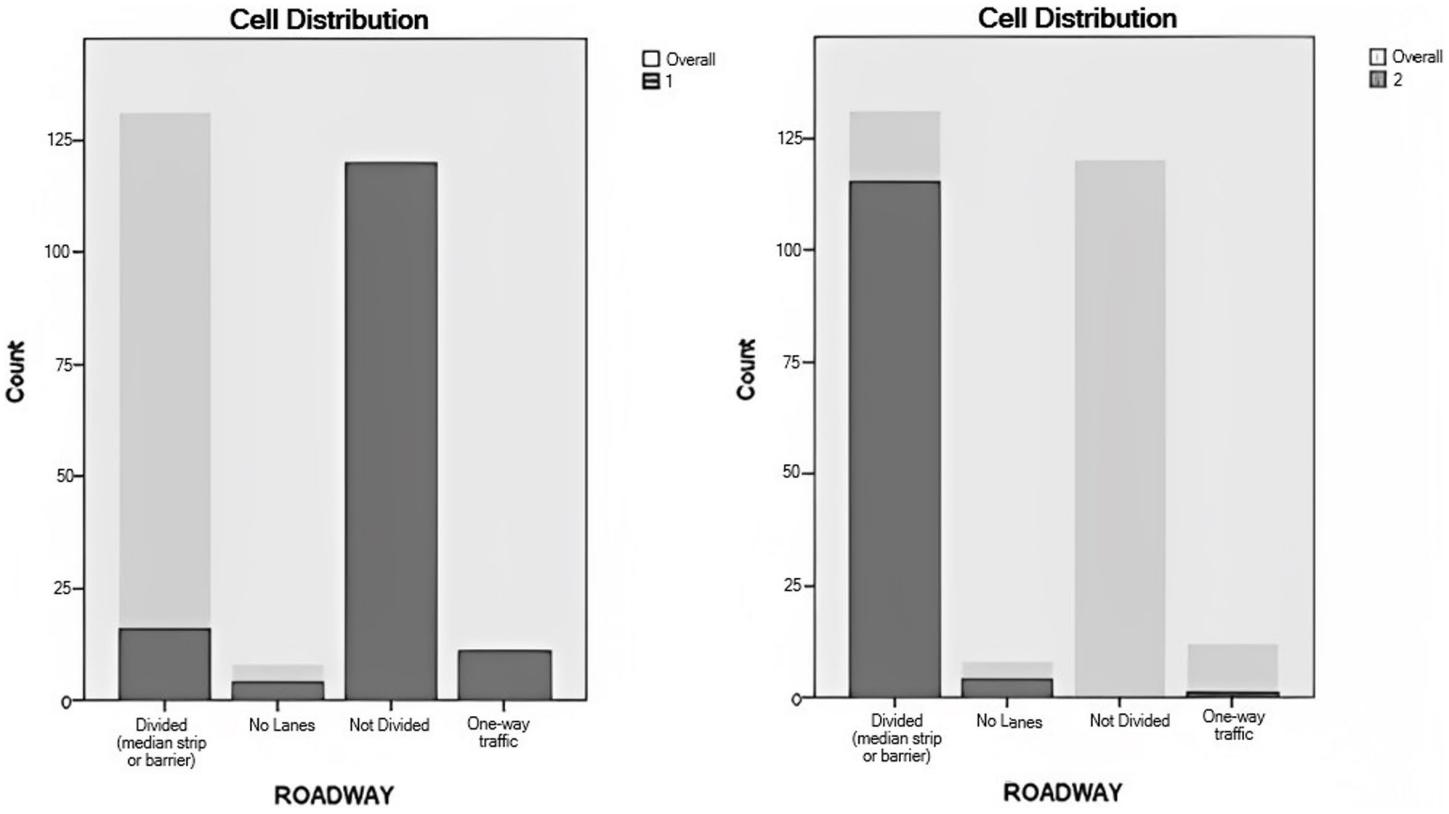
Roadway distribution in clusters.

**Figure 8:**
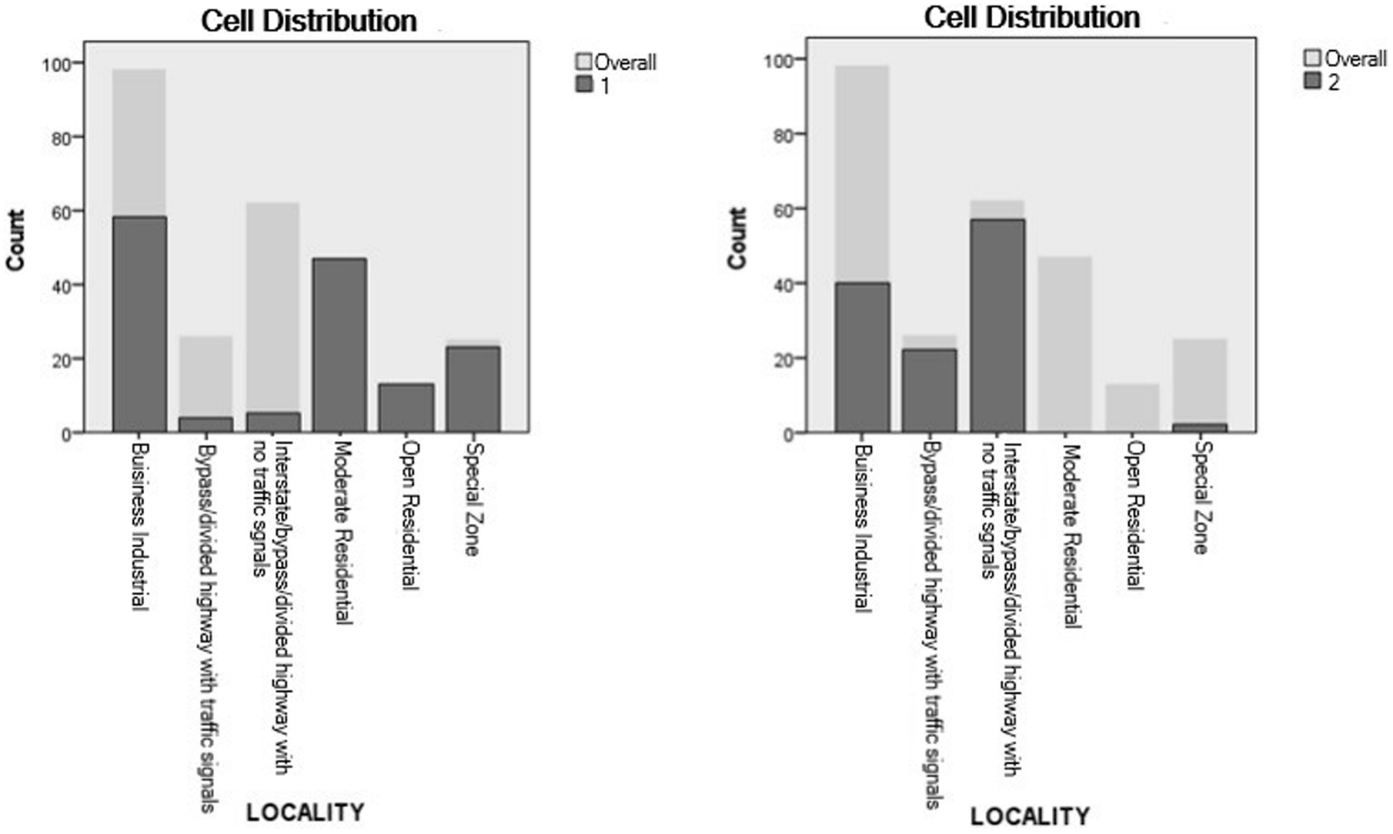
Locality distribution in clusters.

Roadway design was the most important predictor for cluster classification. In Cluster 1, the majority of secondary task occurrences (79.5%) took place on non-divided roads. On the other hand, secondary task occurrences in Cluster 2 were more predominant in divided roads (95.8%). The second most relevant predictor was locality. In Cluster 1, the business/industrial category was the most common locality for engagement (39.07%) and a higher engagement was evidenced in residential areas, both open and moderate, when compared to Cluster 2. For Cluster 2, most engagement occurrences took place in highways with no traffic signals (47.5%), business/industrial localities (32.5%), and highways with traffic signals (18.3%), in that order (Figures 7, 8).

Speed was ranked third in terms of importance. Cluster 1 was characterized by lower speeds with a mean speed of 54.21 while the mean speed for Cluster 2 was higher at 83.35.

**Figure 9:**
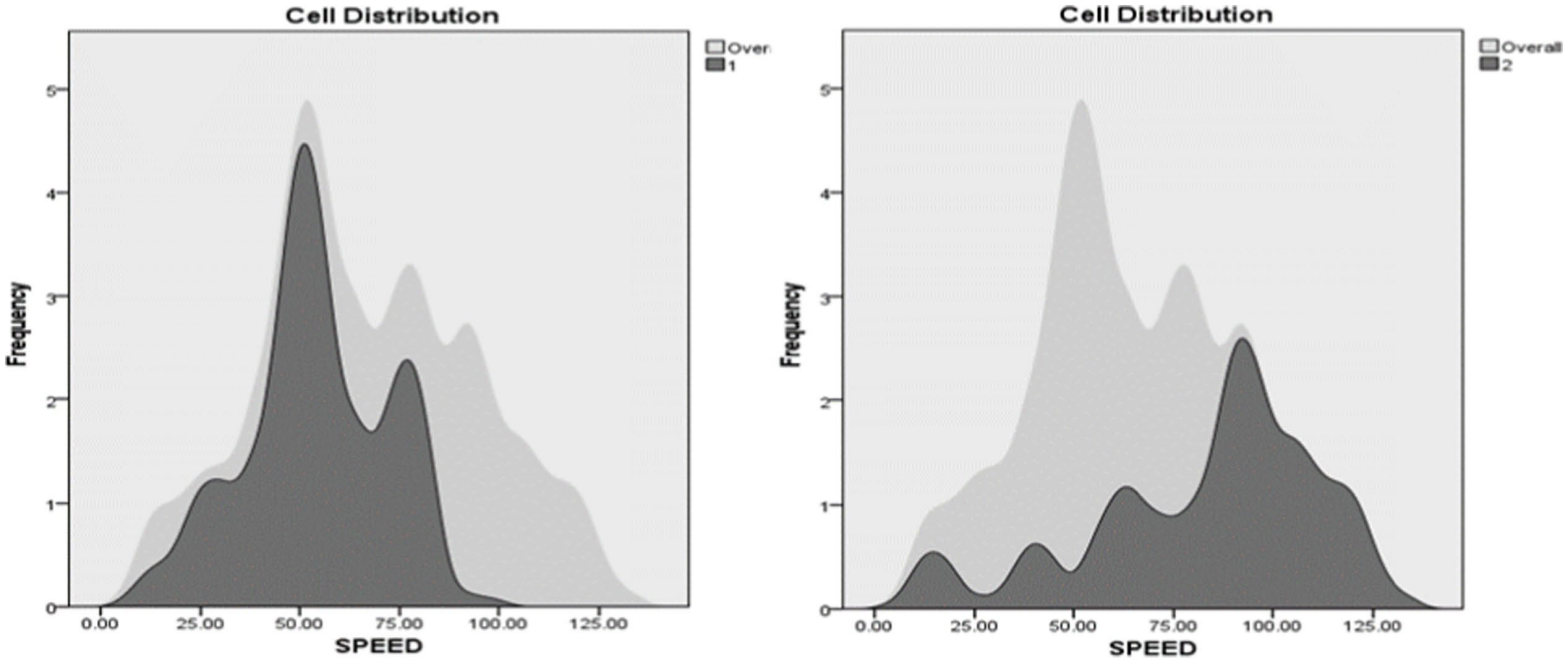
Speed distribution in clusters.

**Figure 10:**
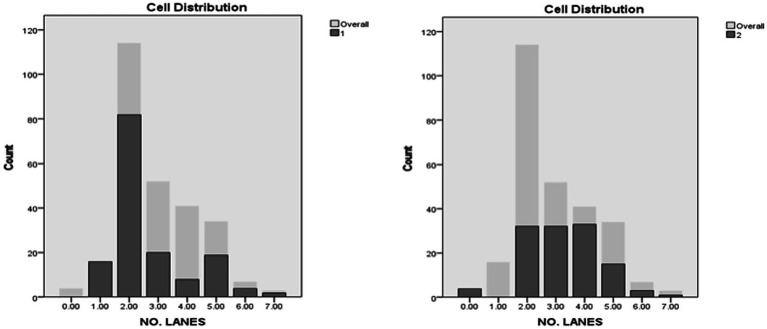
Number of lanes distribution in clusters.

The fourth predictor in importance was the number of lanes. Occurrences in Cluster 1 were markedly higher in 2 lane roads, comprising 54.3% of the total occurrences. The remaining bulk of occurrences was mostly allocated in 3- and 5-lane roads, with around 13.2 and 12.6% of total instances, respectively. Oppositely, Cluster 2 was characterized by higher peaks of engagement between 2- and 4-lanes roads, each configuration accounting for around a quarter of the total occurrences (Figures 9, 10).

**Figure 11:**
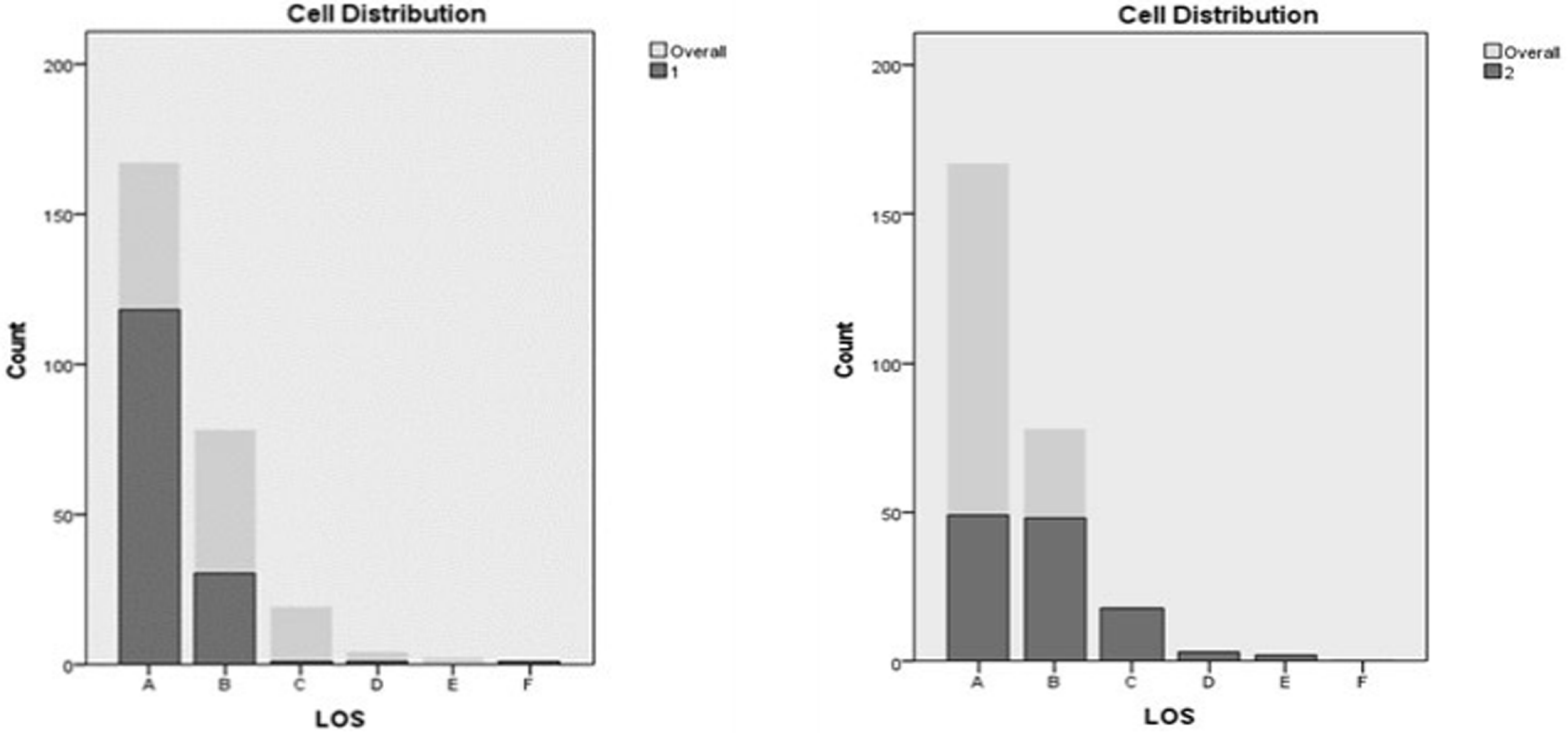
Level of service (LOS) distribution in clusters.

Level of service occupied the fifth position in importance. While secondary tasks occurrences were more common in A and B level of service conditions for both clusters, the distribution was not the same. Cluster 1 contained a markedly higher (78.1%) number of occurrences under level of service A conditions compared to a merely 19.9% of occurrences under B level of service conditions. For cluster 2, the number of occurrences under A and B level of service conditions was fairly similar, with 40.83 and 40%, for levels A and B, respectively. In addition, occurrences for level of service C were markedly higher in Cluster 2 when compared to Cluster 1. Occurrences for levels of service D-F were low for both clusters, however, Cluster 1 contained less occurrences of engagement compared to Cluster 2 (Figure 11).

**Figure 12:**
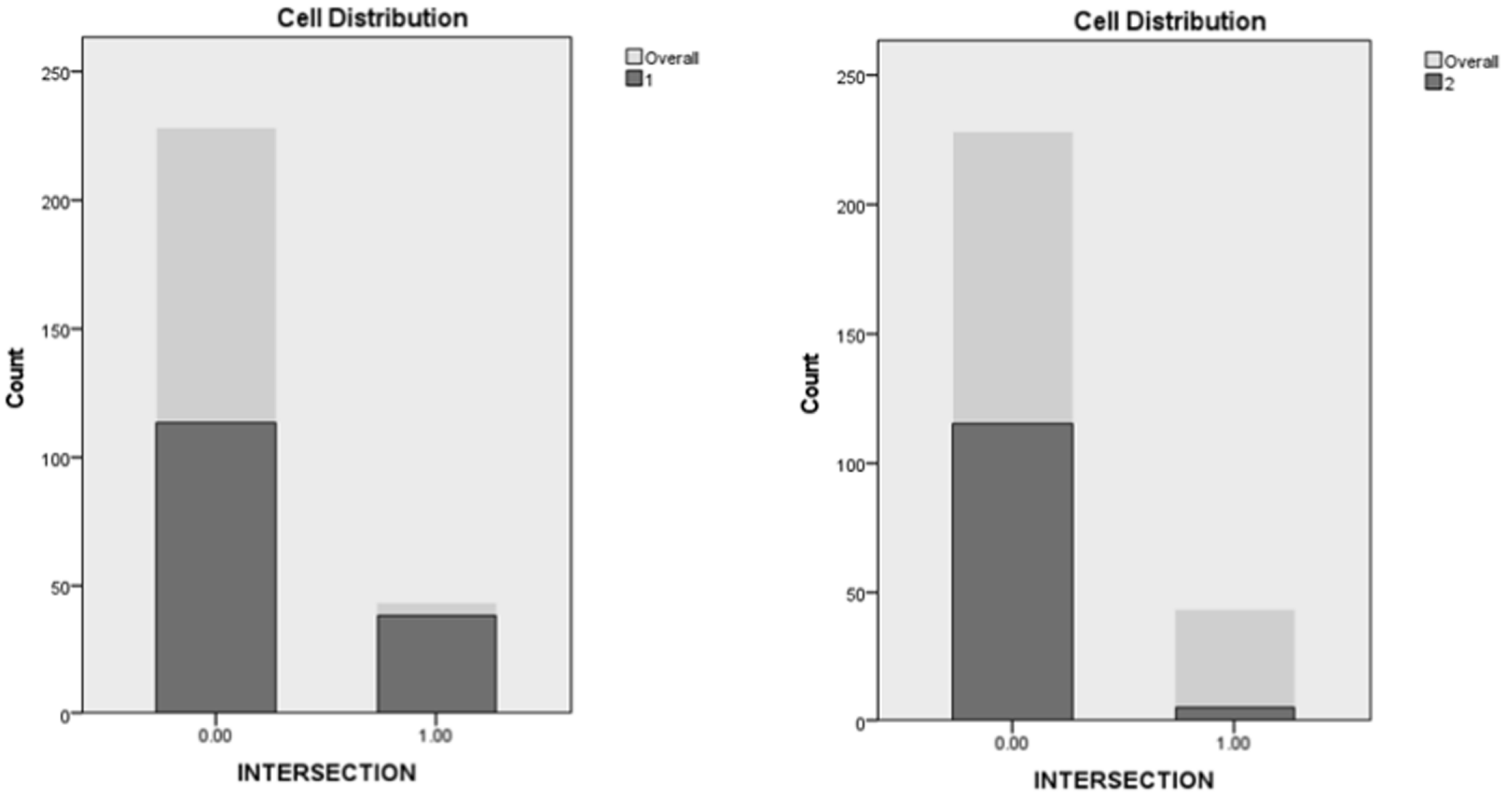
Intersections entered distribution in clusters.

The influence of intersections was ranked as the sixth predictor in importance. Occurrences of engagement in secondary activities at intersections were less common in instances contained in Cluster 2 (4.2%) when compared to instances in Cluster 1 (25.2%; Figure 12).

**Figure 13:**
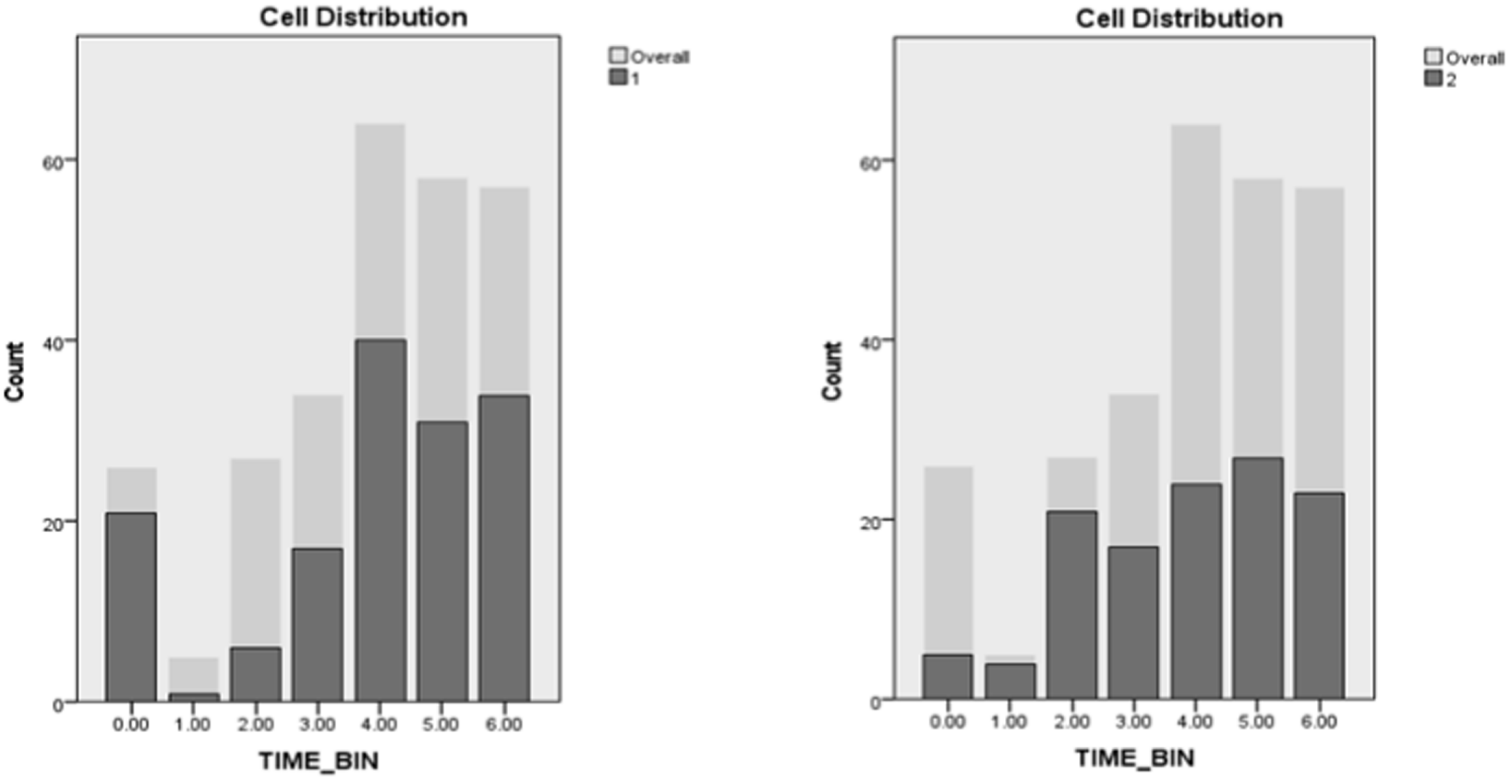
Time bins distribution in clusters.

**Figure 14:**
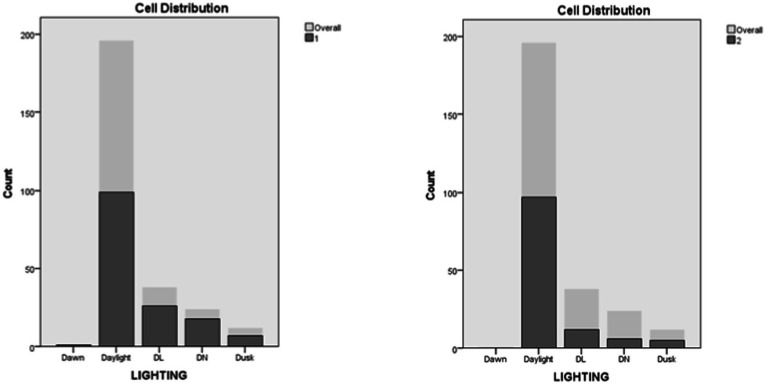
Lighting distribution in clusters.

Time bin and ambient lighting were positioned seventh and eighth in importance among predictors. In Cluster 1, the lowest rates of engagement occurred between 3 AM and 12 PM, with a rising trend. From 12 PM to 3 AM, engagement rates were the highest with a mostly uniform distribution with slight peaks in the 12–3 PM and the 6–9 PM time windows. In Cluster 2, Lower rates of engagement were evidenced between 12 and 6 AM, for the rest of the day (6 AM to 9 PM), engagement rates were higher but mostly uniformly distributed with a slight peak between (3 and 6 PM). As a result, Cluster 1 contained more instances of engagement during darkness when compared to Cluster 2 (Figures 13, 14).

The distribution of secondary tasks among clusters is shown in Table 5. Both mobile phone secondary tasks, texting and holding, were more prominent in Cluster 2 when compared to Cluster 1, however, the difference was not large. The remaining activities were more prominent in Cluster 1, while internal device and passenger interaction were more evenly distributed among the two clusters, dancing and grooming were noticeably more prominent in Cluster 1. None of these differences were deemed significant when conducting maximum likelihood chi-square tests.

**Table 5:** Distribution of secondary tasks in clusters.

Secondary Task	Cluster 1 (%)	Cluster 2 (%)
Mobile phone (Texting)	45.3	54.7
Mobile phone (Holding)	45.5	54.5
Internal device	59.7	40.3
Passenger interaction	54.3	45.7
Grooming	71.4	28.6
Dancing	65	35

## 4. Discussion

This study investigated the relationships between road traffic context of the drivers’ decisions to engage in distracted driving. An initial descriptive analysis found several driving behavioral patterns in relation to contextual variables and secondary task engagement. For instance, higher engagement in secondary tasks was reported during right curves when compared to left curves. A possible explanation for this might be that drivers are under higher attentional demands while driving in left curves due to oncoming traffic and blind spots. Although the rates varied depending on the secondary tasks, interestingly, secondary tasks that require manual interactions such as mobile phone activities and internal device use were more prominently executed in right curves when compared to left curves. On the contrary, passenger conversations were the only task in which engagement was markedly higher in left curves. It is possible that the perceived higher demands of left curves might compel the occupants of the vehicle to engage in conversations to provide input regarding the ongoing driving manoeuvre or the driving context.

Engagement in secondary tasks was also higher while driving uphill compared to driving downhill, which was consistent for all secondary tasks. The most evident explanation for this is that maneuverability requirements and the rapid increase of speed while going downhill discourages engagement in secondary tasks. A naturalistic study by Deng et al. (2019) found greater physical load is required for foot-operated control on downhill segments compared to uphill segments. The study found that pedal force can be regarded as an index that effectively describes the driver’s psychological workload. As such, the continuous speed increase under the action of the force component along the slope direction while driving downhill results in a higher workload in which drivers use more pedal force to avoid loss of directional control.

Another finding that applied for all secondary tasks was a clear trend of decreasing engagement as traffic density increases. Previous research has shown that traffic density increases workload as drivers need to monitor more closely elements of the dynamic traffic conditions such as speed variations, headway distances, traffic flow conditions, changes in lateral position and presence of lane changes (de Waard et al., 2008; Teh et al., 2014). Therefore, it is expected that less resources are available to be allocated for secondary task engagement. Similar results were obtained in a naturalistic study by Gershon et al. (2017) who found that engagement in secondary activities among teens was more prevalent in free flow conditions compared to flow conditions with restrictions. Analysis conducted using data from the Australian Naturalistic Driving Study (ANDS) also showed that secondary task engagement while driving decreased as traffic density increased (Young et al., 2019).

Engagement in secondary tasks while driving was higher during the afternoon periods compared to morning periods. The most common hours for engagement were between 12 and 9 PM. Although, studies tend to differ in the grouping of time bins, a previous observational study by Kidd et al. (2016) also reported higher rates of engagement during the afternoon (11 AM–1 PM) and the evening (4:30–7 PM). In addition, several research efforts focusing solely on mobile phone use while driving have also indicated a prevalence of engagement during the afternoon periods (e.g., Xiong et al., 2014; Sullman et al., 2015). A potential explanation for this is that drivers are using the mobile phone when commuting, to make the experience more enjoyable or useful (Jachimowicz et al., 2021). When the workday is over, individuals may use their mobile phones while commuting as an attempt to replenish the resources lost during the workday, for example by chatting with family or friends or detaching from work by looking at posts or pictures (Ohly and Latour, 2014). Previous research with workers in Italy also demonstrated that work experiences influence phone use during driving commutes (Costantini et al., 2022).

To determine whether there was an association between contextual variables and the types of secondary tasks, Maximum likelihood Chi-square tests were carried out. Associations between roadway design, locality, and speed with types of secondary task were found to be significant. In relation to roadway design, mobile phone use in divided roads was considerably higher when compared to non-divided roads. This is in line with previous findings by Sharda et al. (2019) using the SHRP2 dataset who theorized that the prevalence of engagement in divided roads was the result of the sense of safety given by the existence of barriers shielding the driver from oncoming vehicles in their path. In contrast, grooming and dancing were noticeably higher in non-divided roads when compared to divided roads. Both tasks were also more common on residential areas in comparison with other secondary tasks. A potential explanation is that drivers in residential areas may be near home, which results in poor risk calibration. Familiarity with the road activities results in automaticity and inattention which generally explains why it is more likely that drivers will have a crash closer to home (Charlton and Starkey, 2013). In this case, that familiarity could be linked with a perception of more spared capacity and therefore higher likelihood of engagement.

Regarding localities, for all secondary tasks (except grooming), engagement was more frequent in Business/Industrial localities. Grooming was more common in moderate residential areas, which might be logical as drivers may try to complete grooming tasks after leaving their residencies when pressed for time. Similarly, internal device use in moderate residential was also shown to be higher compared to most secondary tasks which could be explained by drivers setting internal components of the vehicle at the beginning of the trip. In highways with no traffic signals, rates of mobile phone and device use were markedly higher in comparison to tasks such as grooming and dancing. In contrast, highways with traffic signals seemed to favor tasks such as passenger interactions and dancing.

Mobile phone use tasks exhibit similar engagement patterns when compared to the remaining secondary tasks for speeds above 45 km/h. For speeds below 45 km/h, the two tasks exhibit contrasting behavior with lower engagement rates for texting. These results are partially in line with findings from a systematic literature review on mobile phone distraction while driving conducted by Cuentas-Hernandez et al. (2023). While lower engagement at the highest speeds was a common finding, the results for low speeds were dissimilar. Most of the studies reviewed by Cuentas-Hernandez et al. (2023) that considered visual manual mobile phone tasks reported higher engagement rates at low speeds or while the vehicle was stopped. On the contrary, the distribution of instances of engagement for texting and holding activities in the NEST dataset showed the lowest rates of engagement for speeds below 25 km/h.

Passenger interactions, dancing and grooming also share similar speed preferences for engagement when compared to the remaining tasks. Internal device use displayed the most dissimilar engagement preferences when compared with the remaining tasks. Engagement between 20–45 km/h and 65–90 km/h was markedly lower while engagement between 45–70 km/h and 90–110 km/h was markedly higher when compared to all other tasks.

A cluster analysis was conducted to determine which secondary tasks shared similar driving scenarios for execution. Several attentional resource theories suggest that when executing tasks simultaneously, the driver allocates free resources from a shared pool within the tasks. Therefore, driving scenarios sharing similar levels of complexity should allow for a similar number of free resources to be allocated to secondary tasks. It was expected that secondary tasks that share similar resource demands characteristics would be able to be accommodated in driving scenarios with comparable levels of complexity. The less complex the driving scenario, the greater the driver’s ability to accommodate high-demand secondary tasks (Onate-Vega et al., 2020).

The two-step cluster analysis yielded two clusters using eight contextual variables. There were several distinctive characteristics between the two clusters. Driving scenarios in Cluster 2 include some contextual characteristics that have been commonly associated with high demand scenarios. For instance, occurrences in Cluster 2 took place at higher speeds, and were more common in more densely traffic scenarios when compared to Cluster 1. In addition, execution was not as common at intersections, which are often preferred when executing complex secondary tasks due to the momentarily reduction of driving demands while at a standstill. While Cluster 1 occurrences were more prominent in business/industrial locations and residential areas, Cluster 2 occurrences were rarely executed in residential locations. Most instances of engagement in Cluster 2 took place in highways and business/industrial locations.

Another important difference is that occurrences in Cluster 1 were less common within 6 AM and 12 PM and more common between 12 and 3 AM when compared to Cluster 1. Therefore, Cluster 1 contains more instances of secondary tasks performed under dark light ambient conditions. It is possible that lower demand driving scenarios contained within Cluster 1 allowed for execution under dark light ambient conditions while the higher demand scenarios contained in Cluster 2 discouraged execution while driving in dark light ambient conditions.

When considering secondary tasks distribution among clusters, no significant differences were found for secondary tasks between the two clusters. Mobile phone secondary tasks were slightly more prominent in Cluster 2 when compared to Cluster 1. For texting, this suggests that motivation may impaired drivers’ self-regulation processes as the expected lower engagement in texting in high complexity scenarios was not observed. Additionally, as suggested by Oviedo-Trespalacios et al. (2019), texting may be considered by drivers a shorter and less intrusive task which may lower their perceptions of risk.

The remaining activities were more prominent in Cluster 1, but again differences were not found to be significant. These results seem to confirm that scenario-related variables alone only explain a small part of distractions while driving and that larger consideration should be given to task-related and personal-factors. Drivers do indeed use road traffic environment to assess engagement opportunity, but other systemic factors need to be addressed. This research confirms the conclusion by Oviedo-Trespalacios et al. (2018a,b) that self-regulation of mobile phone use depends on the context, the individual and the secondary task at hand.

## 5. Limitations

Limitations associated to the use of the NEST dataset were identified during the data reduction process. For baseline epochs, summary and frame-by-frame data is only available for a 20s time frame, which did not necessarily include the point of initiation of the secondary task. Therefore, only secondary tasks that initiated during the 20s-time window were included for further analysis. Tasks with a shorter execution period were favored for inclusion while tasks with longer execution periods such as phone calls were less likely to register an initiation time during the 20s time frame. As a result, some secondary activities of interest could not be included due to the low quantity of events retrieved. In addition, instances of missing data were encountered in the dataset, mostly impacting time series/frame-by-frame data variables. Ultimately, contextual variables such as weather and road surface condition were also excluded from the analysis as the number of events retrieved during adverse weather conditions (rain, snow, fog) was relatively low.

## 6. Conclusion

This study investigated the relationship between contextual components of the driving task and the decision to engage in driver distraction activities. The analysis was carried out using the NEST dataset derived from the Second Strategic Highway Research Program (SHRP2), the largest to date naturalistic driving study in the United States. Several engagement behavioral patterns were identified. For instance, higher engagement in secondary tasks was reported driving uphill compared to driving downhill, and during afternoons compared to morning periods. In addition, engagement in secondary tasks consistently decreased while traffic density increased. Drivers have demonstrated a preference for engaging in distractions while driving along right curves, compared to left curves.

Significant associations between context and the type of secondary task were found for three variables: roadway design, locality, and speed. In addition, a clustering analysis was conducted to identify secondary tasks that share similar contextual characteristics for execution. The two-step cluster analysis yielded two distinctive clusters with one cluster encompassing scenarios that are associated to higher driving demands compared to the other. No significant differences were found for secondary tasks when considering their distribution among the two clusters. Result suggest that scenario-related variables alone only explain a small part of distractions while driving and that more significant consideration should be given to task-related and personal factors.

## Data availability statement

The SHRP 2 dataset is currently managed by the Virginia Tech Transportation Institute (VTTI) and is made available to support research efforts. As the data for this dataset was obtained from volunteers, it qualifies as Human Subjects Research, and its usage is restricted. Therefore, obtaining access to both the SHRP2 dataset and the NEST dataset is subject to obtaining a data use license. This publication used the dataset with the unique object identifier (DOI): 10.15787/VTT1/OZQBL. The findings and conclusions of this paper are those of the author(s) and do not necessarily represent the views of VTTI, the Transportation Research Board, the National Academies or the Federal Highway Administration.

## Ethics statement

Ethics review and approval was not required in accordance with local legislations and institutional requirements. The study utilized an already existing naturalistic dataset, and the required data use licenses were obtained from the Virginia Tech Transportation Institute (VTTI).

## Author contributions

All authors listed have made a substantial, direct, and intellectual contribution to the work and approved it for publication.

## Funding

This project was funded by a Discovery Early Career Research Award from the Australian Research Council (DE200101079) awarded to OO-T. SC-H, XL, MK, and OO-T also received support from the Motor Accident Insurance Commission (MAIC), Queensland. The views expressed herein are those of the authors and are not necessarily those of the funders.

## Conflict of interest

The authors declare that the research was conducted in the absence of any commercial or financial relationships that could be construed as a potential conflict of interest.

## Publisher’s note

All claims expressed in this article are solely those of the authors and do not necessarily represent those of their affiliated organizations, or those of the publisher, the editors and the reviewers. Any product that may be evaluated in this article, or claim that may be made by its manufacturer, is not guaranteed or endorsed by the publisher.
