# Corrigendum: The impact of road traffic context on secondary task engagement while driving

**DOI:** 10.3389/fpsyg.2024.1465841

**Published:** 2024-08-16

**Authors:** Sandra Cuentas-Hernandez, Xiaomeng Li, Mark J. King, Oscar Oviedo-Trespalacios

**Affiliations:** ^1^QUT Faculty of Health, School of Psychology and Counselling, Queensland University of Technology (QUT), Brisbane, QLD, Australia; ^2^Delft Faculty of Technology, Policy and Management, Department of Values, Technology and Innovation, Delft University of Technology, Delft, Netherlands

**Keywords:** driver distraction, risky behavior, attention, multitask, human factors

In the published article, there was an error in the Data Availability statement. Additional information needs to be added to the Data Availability Statement. The original statement established:

The SHRP 2 dataset is currently managed by the Virginia Tech Transportation Institute (VTTI) and is made available to support research efforts. As the data for this dataset was obtained from volunteers, it qualifies as Human Subjects Research, and its usage is restricted. Therefore, obtaining access to both the SHRP2 dataset and the NEST dataset is subject to obtaining a data use license.

The correct Data Availability statement appears below.

## Data Availability

The SHRP 2 dataset is currently managed by the Virginia Tech Transportation Institute (VTTI) and is made available to support research efforts. As the data for this dataset was obtained from volunteers, it qualifies as Human Subjects Research, and its usage is restricted. Therefore, obtaining access to both the SHRP2 dataset and the NEST dataset is subject to obtaining a data use license. This publication used the dataset with the unique object identifier (DOI): 10.15787/VTT1/OZQBL. The findings and conclusions of this paper are those of the author(s) and do not necessarily represent the views of VTTI, the Transportation Research Board, the National Academies or the Federal Highway Administration. In the published article, there was a redaction error in the abstract where it incorrectly stated higher engagement rates in left curves compared to right curves, contrary to the paper's findings. A correction has been made to **the Abstract**, *Results Subsection, Paragraph 1*. This sentence previously stated: “The exploratory analysis revealed interesting behavioral trends among drivers, with higher engagement rates in left curves compared to right curves, while driving uphill compared to driving downhill, in low-density traffic scenarios compared to high-density traffic scenarios, and during afternoon periods compared to morning periods.” The corrected sentence appears below: “The exploratory analysis revealed interesting behavioral trends among drivers, with higher engagement rates in right curves compared to left curves, while driving uphill compared to driving downhill, in low-density traffic scenarios compared to high-density traffic scenarios, and during afternoon periods compared to morning periods.” The authors apologize for these errors and state that this does not change the scientific conclusions of the article in any way. The original article has been updated.

